# Case Report: Endovascular electrothrombosis treatment for carotid cavernous fistula caused by ruptured primitive trigeminal artery aneurysm

**DOI:** 10.3389/fsurg.2025.1559977

**Published:** 2025-03-21

**Authors:** Yuhui Wan, Zengjing Cheng, Ziyan Lu, Dehong Yang, Zhaoliang Li, Kai Yang, Binglin Chen, Ailin Chen, Qing Zhu

**Affiliations:** ^1^ Department of Neurosurgery, Second Affiliated Hospital of Soochow University, Suzhou, Jiangsu, China; ^2^ Department of Neurology, Second Affiliated Hospital of Soochow University, Suzhou, Jiangsu, China

**Keywords:** persistent primitive trigeminal artery, intracranial aneurysm, carotid cavernous fistula, endovascular coiling, electrothrombosis

## Abstract

Aneurysms of a persistent trigeminal artery (PTA) are exceptionally uncommon, and their rupture leading to a carotid-cavernous fistula (CCF) is even more extraordinary. The contemporary management of CCF predominantly revolves around endovascular approaches, with a variety of techniques such as detachable balloons or coils, coil embolization augmented with Onyx adhesive, and stent grafting. Herein, we report a successful intervention in a patient with a CCF instigated by a ruptured PTA aneurysm, employing a combination of detachable coils and endovascular electrothrombosis, yielding favorable outcomes.

## 1 Introduction

The persistent trigeminal artery (PTA), an embryonic vestige linking the internal carotid artery (ICA) to the basilar artery (BA), is the most frequently encountered primitive connection between the anterior and posterior cerebral circulations in adults, typically manifesting unilaterally (1). Generally asymptomatic, PTA is often discovered incidentally through imaging, rarely requiring intervention. However, large-scale cerebral angiography studies indicate its incidence ranges only from 0.1% to 0.6% (2). Notably, about 25% of PTA instances are associated with cerebrovascular anomalies due to the artery's developmental peculiarities and distinct hemodynamics (1, 3, 4). These anomalies include intracranial aneurysms (IA), arteriovenous malformations, CCF (5), and moyamoya disease (6), with IAs being notably rare at 3% occurrence (7), and CCF formation following rupture being extraordinarily uncommon. This paper discusses a rare case of a CCF resulting from a ruptured PTA aneurysm, which was effectively treated using coil embolization alongside endovascular electrothrombosis, supplemented by a thorough review of relevant literature.

## 2 Case report

**Figure 1:**
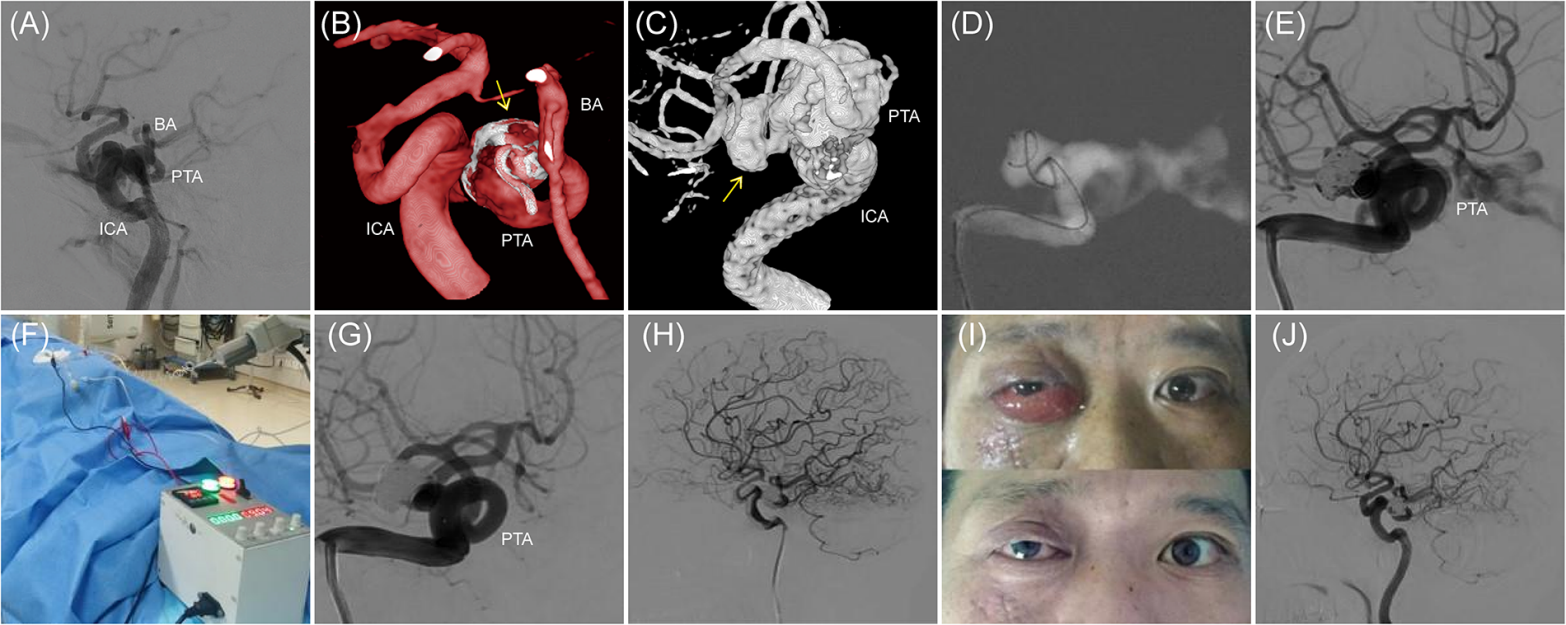
The case of a ruptured right PTA aneurysm leading to a CCF. **(A–C)** Right ICA injection, lateral and three-dimensional reconstruction, working angle, showing the CCF. The abnormal arteriovenous shunt is located at the top of the right PTA aneurysm. The arrow points to the aneurysm. **(D)** Navigation of 2 Echelon10 microcatheters through the fistula into the PTA aneurysm sac. **(E)** After filling with 9 detachable coils, control angiography reveals that the abnormal arteriovenous shunt is still evident. **(F)** The homemade electrical connection device is used to create an electrical circuit for electrothrombosis. **(G,H)** After 7 min of applying 9 V voltage and 1 mA current, the abnormal arteriovenous shunt completely disappears, and the ICA and PTA remain intact. **(I)** The patient's facial appearance before and one week after endovascular procedure. **(J)** Follow-up angiography 6 months later shows the disappearance of the original right CCF's abnormal arteriovenous shunt, with a well-preserved ICA and PTA and good distal blood flow.

A 42-year-old male presented with a medical history significant for head trauma occurring over two months prior and subsequent proptosis of the right eye noted for three weeks. The physical examination revealed an evident protrusion of the right eye, accompanied by conjunctival congestion and edematous swelling. Digital subtraction angiography (DSA) established a diagnosis of a partial CCF on the right, concomitant with a PTA. The proximal segment of the PTA demonstrated aneurysmal dilatation (dome size 12.7 mm × 10.5 mm × 6.1 mm, neck width 2.4 mm), with the fistula's orifice located at the aneurysm's pinnacle, precipitating a retrograde flow into the superior ophthalmic vein of the right side and the cavernous sinus of the contralateral hemisphere (Figures 1A–C). Under general anesthesia, access to the right femoral artery was established, and a Terumo 6F sheath (Terumo, USA) was introduced. Then, a 5F MPA diagnostic catheter (Cordis, USA) was advanced over-the-wire (0.035 guidewire, Terumo, USA) to navigate a 6F guiding catheter (Envoy, Cordis, USA) at the C2 vertebral level of the right internal carotid artery. Two microcatheters (Echelon10, eV3, USA) were navigated over a microguidewire (Transend Soft Tip 0.014, Boston Scientific, USA) through the fistula into the cavernous sinus by sequence, whose tip was remolded to “J” and “Z” shape by high temperature steam respectively (Figure 1D). Subsequently, a sequence of Axium coils (ev3, USA) of varying dimensions—including 3D 14 × 40, 12 × 40, and multiple 10 × 30, followed by 2D 9 × 30, 3D 8 × 30, 7 × 30, and finally 4 × 8—were sequentially deployed. However, subsequent angiography disclosed persistent, significant arteriovenous shunting at the fistula site (Figure 1E). Before the detachment of the ultimate pair of coils from the microcatheters, a specialized negative electrode connector (tailored for Neurointerventional Vascular Electrothrombosis Treatment, patent application No. 202221108924.X) was percutaneously positioned in the right groin's musculature. This apparatus was interfaced with a proprietary electropower unit (Portable Adjustable Medical Electrothrombosis Box, patent No. ZL202122348511.0), serving as the negative pole. Concurrently, the distal extremity of the coil delivery pusher was affixed to the positive electrode. For seven minutes, a controlled application of 9 V and 1 mA was executed (Figure 1F). Follow-up angiography demonstrated the total cessation of the abnormal arteriovenous shunting at the fistula location, accompanied by augmented forward blood circulation in both the ICA and the PTA, thus boosting distal ICA perfusion (Figures 1G,H). In light of these favorable outcomes, the coils were securely detached, the microcatheters extracted, and the vascular sheath carefully removed. Local compression was administered and suitable dressing techniques were employed. Postoperatively, the patient presented no new neurological deficits, and the conjunctival edema fully resolved within a week (Figure 1I). Six months post-procedure, a follow-up examination confirmed the absence of abnormal arteriovenous shunting in the initially affected right-sided CCF. The PTA preserved its structural integrity, and the distal blood flow sustained its optimality (Figure 1J).

## 3 Discussion

### 3.1 Anatomy and classification

Among the embryonic cervical-carotid anastomoses, the trigeminal artery (TA) is the most prominent and exhibits the longest duration of persistence. With embryonic development advancing to the stage of approximately 11.5–14 mm, the posterior communicating artery (PCoA) begins to form, thereby marking the start of the TA's regression process (1, 8, 9). If the TA fails to regress completely during the transition to adulthood, it becomes a PTA. Studies indicate that hemodynamic irregularities could be a primary factor influencing this developmental anomaly. Lasjaunias and Berenstein elucidated the persistence of the PTA, highlighting its crucial function in preserving hemodynamic balance across anterior and posterior circulations. This mechanism compensates for the insufficient blood flow from the bilateral vertebral arteries (VA) (10).

Anatomically, PTA anatomically arises from the ICA and courses posteriorly and laterally alongside the trigeminal nerve, connecting with the BA. It commonly originates at the posterior curve or lateral aspect of the cavernous segment of the ICA (11, 12). Various classification methods exist for the PTA, among which Saltzman's classification is particularly notable (13). Type 1 PTA extends to the distal anterior inferior cerebellar artery (AICA) and the proximal vertebrobasilar junction, potentially indicating developmental issues in the proximal BA and a possible absence of the ipsilateral posterior communicating artery (PCoA). In contrast, Type 2 PTA is typically free of developmental deficits, with its distal end reaching the proximal anterior superior cerebellar artery (ASCA), which it supplies, while the posterior cerebral artery (PCA) primarily derives its blood supply from the PCoA. From a clinical treatment perspective, Saltzman's classification significantly reflects the varying vascular territories supplied by the PTA, thereby enhancing its clinical relevance.

### 3.2 Formation and treatment

Davis first reported the coexistence of a PTA and an IA in 1956 (14). Cerebral angiography studies indicate that unilateral PTA aneurysms occur in approximately 0.1%–0.6% of cases (2), and bilateral cases are exceedingly uncommon (15). In an 18-year review of the literature, Cloft found that the prevalence of IA was around 3% among 34 PTA cases, comparable to the 3.7% in the general population (7). O'uchi E and colleagues analyzed 16,415 cases spanning three years, revealing a similar IA incidence of 3.9% (2), with 14%–32% being PTA-related (16). Anomalies in the medial layer of the vascular wall predispose PTA to IA formation and localized ruptures. Previous report revealed that PPTA developmental defects in the middle layer of the vessel wall and its specific anatomical alignment create a basis for congenital vulnerability, often serving as a prerequisite for aneurysm formation (9). Additionally, most PTA aneurysms are located at the bifurcation of the cavernous segment of the ICA and the PTA (17). This vulnerability may be attributed to the direct connection of the high-flow ICA to the BA, which creates a flow 'short-circuit effect' that results in aneurysm formation and local rupture at the junction of the cavernous segment of the ICA with the PTA due to abnormally high wall shear stress. Reports suggest a 50% rupture rate for PTA aneurysms, with half leading to CCF, 45% to SAH, and 5% to recurrent epistaxis (18).

Treatment strategies for PTA aneurysms ought to be tailored according to their vascular territory, guided by Saltzman's classification. Preservation of Type 1 PTA is critical; if infeasible, arterial bypass may be necessary to maintain posterior circulation. Vigilant monitoring of Type 2 PTA is imperative due to its potential role in recurrence, with occlusion as an option if required. In the case under discussion, DSA showed that the PTA supplied the upper segment of the BA, classifying it as a Saltzman Type 1.

A PubMed search utilizing the keywords “persistent primitive trigeminal artery,” “aneurysm,” and “carotid cavernous fistula” retrieved 11 pertinent studies (Table 1). Of the 11 patients profiled, 2 exhibited a documented trauma history (18.2%), with the remainder presenting spontaneous cases (81.8%). PTA aneurysms or breaches at vulnerable sites within the PTA vascular structure were identified as the primary etiologies (28). Typical clinical presentations encompassed proptosis, conjunctival engorgement, cranial murmurs, and double vision. Due to the structural vulnerability of the PTA, the formation and subsequent rupture of PTA aneurysms often lead to spontaneous CCF (29). Traumatic CCF typically occurs when external forces exert pressure on the head during trauma, causing the PTA to endure maximum shear stress during acceleration or deceleration injuries (29, 30), which may result in the rupture of the proximal segment of the PTA. Therapeutic interventions for PTA aneurysms comprise microsurgical clipping and endovascular coiling. Microsurgical clipping is optimally employed for IAs with narrow necks and diminutive sizes, featuring readily accessible anatomical structures. Nonetheless, the frequent positioning of PTA aneurysm necks within the cavernous sinus renders surgical intervention complex and hazardous. While Enomoto and colleagues previously achieved success in treating several cases through ICA ligation (8), such an approach is heavily contingent upon the Circle of Willis' compensatory capabilities and may elevate the risk of flow-related IAs over time. Consequently, in light of considerable innovations in interventional methodologies since the 1990s, endovascular therapy has emerged as the favored modality for managing PTA aneurysms (31). This paradigm incorporates procedures like balloon occlusion, coiling, and the synergistic application of coils with Onyx embolization. Initially, detachable balloon occlusion was the treatment of choice (28). However, the convoluted nature of vascular flow and pathways may at times impede balloon navigation through the fistula into the cavernous sinus (32, 33), posing a risk of recurrence. Among the reviewed cases, a mere two instances of successful detachable balloon occlusion with positive prognoses are reported. Liu and associates were the pioneers in documenting the employment of detachable coils for the resolution of CCF secondary to ruptured PTA aneurysms, with beneficial results (21). While coils provide superior controllability compared to balloons, the typically larger size of PTA aneurysms necessitates the use of an increased coil count for effective embolization. Moreover, the exclusive use of coils cannot assure the complete eradication of abnormal arteriovenous shunting, and the mass effect resulting from overfilling presents a significant resolution challenge. Fan et al. devised a surgical method that employs a hybrid of coils and Onyx glue, reducing the necessity for multiple coils and facilitating targeted treatment (25). However, Onyx glue's limited controllability poses a challenge, necessitating protective measures for the ICA and BA to prevent accidental embolization (34).

**Table 1:** Summary of the 11 previous reported cases of PPTA aneurysm.

Author (Year)	Age (yrs), Sex	Symptoms	Course	Treatment	Result
Enomoto et al. (1977) (8)	42, F	Ocular disorder	Spontaneous	ICA ligation	Cure, mild ophthalmoplegia
Flandroy et al. (1987) (19)	35, M	Ocular disorder, bruit	Traumatic	Balloon	Cure
Guglielmi et al. (1990) (20)	20, M	Ocular disorder	Traumatic	Refused	Unresolved.
Liu et al. (2009) (21)	55, F	Ocular disorder, bruit	Spontaneous	Coil	Cure
Qian et al. (2009) (22)	62, F	Ocular disorder, bruit	Spontaneous	Balloon	Cure
Kim et al. (2010) (23)	42, F	Ocular disorder	Spontaneous	Coil	Cure
Yoshida et al. (2011) (24)	60, F	Ocular disorder	Spontaneous	Coil	Cure
Diana et al. (2019) (18)	61, F	Ocular disorder, bruit	Spontaneous	Coil	Cure
Fan et al. (2019) (25)	64, F	Ocular disorder	Spontaneous	Coil and Onyx glue	Cure
Shiomi et al. (2021) (26)	69, F	Ocular disorder, bruit	Spontaneous	Coil	Cure
Sun et al. (2022) (27)	55, F	Ocular disorder	Spontaneous	Coil and Onyx glue	Cure

In the case under discussion, a CCF manifested subsequent to the rupture of the aneurysm of the PTA within the cavernous sinus. The fistula, situated at the proximal segment of the PTA, demonstrated partial arteriovenous shunting. Given these characteristics, conventional treatment modalities, including microsurgical clipping and detachable balloon embolization, were deemed unsuitable. As a result, detachable coil embolization emerged as the treatment of choice. Preoperative computed tomography angiography (CTA), processed using Mimics 8 software (Materialise, USA), estimated the aneurysm volume at 675.56 mm^3^. The embolization procedure involved a dual microcatheter strategy, with a total of nine coils deployed for aneurysm occlusion. Post-embolization, the aneurysm volume was diminished to 275.75 mm^3^, yielding a packing density of 40.82%. Such a degree of embolization can be classified as considerable. Nonetheless, angiographic examination revealed substantial persistent arteriovenous shunting with elevated blood flow velocity. Although Onyx glue was contemplated as an adjunctive measure, achieving an efficacious seal at the aneurysm neck with the adhesive was particularly challenging in this anatomical context. Moreover, the inherent unpredictability of the embolization technique spurred apprehension regarding potential modifications in PTA hemodynamics or inadvertent embolization. To circumvent these obstacles, adjunctive endovascular electrothrombosis was utilized along with coil embolization. This integrative method facilitated the comprehensive occlusion of the fistula, concurrently conserving the patency of the PTA. This strategy effectively circumvented the mass effect linked to excessive coil deployment and attenuated the hazard of non-target embolization, a noted concern with Onyx glue application.

The novel technique of endovascular electrothrombosis was originated by Jiang Yuhua et al. at Beijing Tiantan Hospital, introduced to the medical field in 2016. Its initial application, in concert with bare metal microguidewires, addressed diminutive aneurysms nestled within the perforating arteries of the basilar artery. This approach was particularly advantageous in scenarios where access to these formidable loci was unattainable by the microcatheter tip (35). An exhaustive exploration of databases such as PubMed and CNKI has yielded merely seven clinical case reports pertinent to this technique (36–39). Commencing in 2018, our institution has broadened the utilization of this method, augmenting the instantaneous complete occlusion rates of cystic aneurysms still presenting with contrast filling post-coil embolization. The deployment of this expanded application has resulted in superlative treatment outcomes (37). Jiang and his colleagues have conceptualized a theoretical framework for this technique, postulating that its microcosmic mechanism is underscored by two pivotal processes: thrombogenesis and thrombus organization (38). Preliminary experiments at our institution have disclosed that electrification of the microguidewire tip, serving as an anode, draws in negatively charged blood constituents such as erythrocytes, thrombocytes, and leukocytes, thus catalyzing thrombogenesis. In tandem, plasma electrolysis at the microguidewire tip engenders bubble formation, furnishing a scaffolding for the accruing thrombus in the vicinity of the wire's tip. Additionally, the electrical current activity at the microguidewire tip generates an electrothermal effect, which causes denaturation and reorganization of the existing thrombus. Consequently, this process culminates in the creation of a more stable thrombotic structure. These findings robustly advocate for the feasibility and efficacy of this method for intravascular treatments designed to induce thrombosis (39). Physics research has clarified that the intensity of current activity on metal surfaces is inversely correlated with the surface area of the metal submerged in a liquid medium. This crucial insight implies that enlarging the metal's contact surface area within a liquid can efficaciously disperse current activity, thus augmenting the safety of intravascular electrothrombosis procedures. Adopting a pioneering approach, our institution has implemented the use of mechanically detachable bare metal coils (specifically, Axium coils by ev3, USA) for intravascular electrothrombosis, boasting several significant advantages: (1) These coils provide an extensive contact surface area with the blood within the aneurysm sac, significantly enhancing thrombosis efficiency. The dispersion of current activity across the metal surface is broader compared to the confined tip of a microguidewire, leading to an improved safety profile. (2) The coils adopt a structure comparable to “reinforced concrete” within the aneurysm sac, bolstering the stability of the embolic material. (3) This innovative approach effectively mitigates the potential risk of reperfusion or distal embolization that may occur during the thrombus removal process post-electrothrombosis with microguidewires.

The risks associated with electrothrombosis warrant discussion. Current research indicates that the safety profile of electrothrombosis primarily depends on the unique characteristics of various guidewires and coils, as well as electrical parameters (40). Increasing the voltage can enhance the efficacy of electrothrombosis (37), but excessively high voltages can pose considerable risks, including aneurysm rupture, excessive thrombus formation, and occlusion of the parent artery. Our research team conducted *in vivo* simulated electrothrombosis experiments involving New Zealand white rabbits, revealing that elevated voltages increase the risk of adverse events associated with electrothrombosis, with the presence of gas identified in pathological specimens following the electrothrombosis of experimental aneurysms (39). Nonetheless, an appropriate voltage can still achieve satisfactory thrombotic effects within an acceptable safety range. Furthermore, studies have shown that prolonging the duration of electrical activation at an appropriate voltage can enhance the effectiveness of electrothrombosis without elevating safety risks (39). Additionally, while different microguidewires exhibit varying resistance and physical properties, these differences do not appear to affect the efficacy of electrothrombosis (41).

Drawing from our institution's comprehensive experience, we have established that a voltage of 9 V constitutes a relatively safe threshold for clinical application. Extending the duration of electrical stimulation can achieve comparable thrombosis efficiency while ensuring an elevated level of safety. For this particular case, parameters of 9 V/1 mA were employed. After 7 min of continuous electrical stimulation, complete occlusion of the PTA aneurysm sac was achieved. It is worth noting that the extended duration of electrical stimulation, relative to prior cases of saccular aneurysm occlusion, likely results from the PTA aneurysm's larger size and the increased blood flow within the aneurysm sac, consequent to the associated CCF. To our knowledge, this is the first report on electrothrombosis for CCF caused by ruptured primitive TPA. However, a comprehensive validation of these outcomes necessitates long-term follow-up. This study has several limitations. The safety of electrothrombosis has not been thoroughly established. Although some validation exists from animal models, there is a notable lack of large-scale, multicenter randomized controlled trials to provide robust clinical evidence.

## 4 Conclusion

CCFs secondary to ruptured PTA aneurysms are exceedingly rare, with endovascular intervention being the preferred treatment modality. Surgical intervention should be customized based on the PTA's blood supply extent. Unlike traditional endovascular therapies, the synthesis of mechanical detachable bare metal coil embolization with electrothrombosis offers an innovative method to manage CCF. This technique proffers multiple advantages such as cost-efficiency, diminished mass effect of embolic agents, and reduced risk of complications related to medical glue utilization in embolization. Future studies, including laboratory research and clinical trials, could further substantiate the efficacy and safety of this method. Consequently, this could facilitate the broader implementation of this technique in clinical settings.

## Data Availability

The original contributions presented in the study are included in the article/Supplementary Material, further inquiries can be directed to the corresponding author.
